# VLX1570 regulates the proliferation and apoptosis of human lung cancer cells through modulating ER stress and the AKT pathway

**DOI:** 10.1111/jcmm.17053

**Published:** 2021-12-01

**Authors:** Juan Wang, Tongde Du, Ya Lu, Yan Lv, Yuxin Du, Jianzhong Wu, Rong Ma, Chenxin Xu, Jifeng Feng

**Affiliations:** ^1^ The Affiliated Cancer Hospital of Nanjing Medical University Jiangsu Cancer Hospital Jiangsu Institute of Cancer Research Nanjing China; ^2^ Research Center for Clinical Oncology Jiangsu Cancer Hospital Jiangsu Institute of Cancer Research The Affiliated Cancer Hospital of Nanjing Medical University Nanjing China

**Keywords:** AKT pathway, apoptosis, ER stress, lung cancer, proliferation, VLX1570

## Abstract

The ubiquitin‐proteasome system (UPS) possesses unique functions in tumorigenesis and has great potential for targeting tumours. The effectiveness of inhibitors targeting UPS in solid tumours remains to be studied. We screened a library of inhibitors targeting the ubiquitination system and found the highly potent, low‐concentration inhibitor molecule VLX1570 that specifically killed lung cancer cells. VLX1570 is an inhibitor of deubiquitinating enzyme activity and has also shown potential for preclinical cancer treatment. We found that VLX1570 significantly inhibited lung cancer cells proliferation and colony formation. VLX1570 induced a G2/M cell cycle arrest by downregulating CDK1 and CyclinB1. Moreover, VLX1570 significantly promoted the mitochondrial‐associated apoptosis. Mechanistically speaking, VLX1570 activated the PERK/IRE1/ATF6 pathway and induced ER stress in lung cancer cell lines. The inhibition of ER stress by tauroursodeoxycholic acid sodium or 4‐phenylbutyric acid enhanced VLX1570‐induced apoptosis. In addition, VLX1570 treatment led to the inactivation of Akt signalling and inhibited the proliferation of lung cancer cells by downregulating the Akt pathway. Moreover, combined treatment with VLX1570 and Afatinib or Gefitinib induced synergistic anti‐lung cancer activity. Our present study demonstrated a novel therapy using VLX1570, which inhibited the proliferation and increased apoptosis in human lung cancer.

## 1 INTRODUCTION

Lung cancer is one of the most frequently occurring cancers in the world, ranking second in terms of incidence rate but having the highest mortality rate.^1^ Although therapeutic strategies have been developing over recent years, new treatment methods are still urgently needed.

A diverse set of cellular processes such as cell cycle progression, DNA repair, metabolism and cell survival are dynamically controlled by the synthesis and degradation of protein regulators. The regulated degradation of proteins is controlled mainly by the ubiquitin‐proteasome system (UPS) in eukaryotic cells.^2^ The UPS primarily comprises an E1, E2 and E3 ubiquitin ligase, the proteasome and deubiquitinating enzymes. The proteasome is a large multi‐subunit proteolytic complex that specifically degrades ubiquitin tagged proteins into small peptides.^3^ First, an E1‐activating enzyme activates the 76 amino acid ubiquitin protein which is the ATP‐dependent activation. Second, by the formation of a thioester bond, active ubiquitin is then transferred to an E2 conjugating enzyme through a *trans*‐esterification reaction.^4^ Finally, an E3 ligase enzyme recognizes target proteins for ubiquitination and determines substrate specificity, facilitating the formation of a covalent isopeptide bond and promoting protein ubiquitination. DUBs are a kind of proteases that antagonize the modification mediated by E3 ubiquitin ligases, and they can remove a ubiquitin moiety from a ubiquitylated substrate.^4^

The UPS has been demonstrated to play vital roles in the modulation of tumorigenesis, and many molecular targeted drugs that target the UPS have been developed to combat cancer.^5^ Bortezomib is a peptide boronate that inhibits the activity of the 20S proteasome, resulting in the accumulation of defective proteins. The experiments and clinical development demonstrating this allowed Bortezomib to get subsequent approval to treat relapsed or refractory mantle cell lymphoma combined with melphalan and prednisone (MP regimen).^6^ Although Bortezomib has resulted in success in the treatment of multiple myeloma, the majority of patients treated with Bortezomib have been shown to develop drug resistance and relapse.^7^ The large effort required in the research community to identify additional Inhibition of deubiquitinase (DUB) inhibitors is thus extremely important. Undoubtedly, DUB activity is a promising strategy for cancer therapy.^4^ VLX1570 targets the Ub‐USP14 or Ub‐UCHL5 conjugates and is a reversible non‐selective competitive inhibitor of ubiquitin peptidase 14 (USP14) and ubiquitin carboxyl‐terminal hydrolase 5 (UCHL5) in multiple myeloma.^5^ VLX1570 has been shown to have significant anti‐cancer efficacy with high potency and solubility. Currently, a phase 1/2 trial is ongoing to evaluate the tolerability and efficacy of VLX1570 with relapsed or refractory multiple myeloma patients.^4^

The primary function of the endoplasmic reticulum (ER) is the synthesis of secretory and transmembrane proteins to the Golgi compartment. In addition, the ER is also responsible for the degradation of misfolded/unfolded proteins and the modification of specific proteins.^8^ Any alterations, for example, in chronic inflammation, tumorigenesis, or pathogen infection, can lead to the accumulation of unfolded/misfolded proteins in the ER, which causes disorder of the ER homeostasis and can trigger the expression of chaperones, such as GRP78 (eg 78 kDa glucose‐regulated protein [GRP78]), calreticulin and calnexin.^9^ ER stress utilizes three signalling pathways, including pathways downstream of transcription factor 6 (ATF6), inositol‐requiring enzyme 1 (IRE1) and protein kinase RNA‐like ER Kinase (PERK).^10^ Among the different mechanisms that have been described to underlie the cytotoxicity of deubiquitinase inhibitors in tumour cells, induction of ER stress and the unfolded protein response (UPR) is generally accepted to play an important role. ER stress performs dual functions, either promoting cell survival or triggering cell death, depending on the imbalance between ER protein‐folding load and capacity.^11^, ^12^ Moderate ER stress promotes cancer cell survival and increases chemotherapeutic resistance; however, severe ER stress leads to cancer cell apoptosis.^13^ Previous studies have revealed that VLX1570 induces ER stress in colon carcinoma,^14^ melanoma cells,^15^ Waldenstroms macroglobulinaemia,^16^ prostate carcinoma,^17^ hepatocellular carcinoma cells^18^ and acute lymphoblastic leukaemia.^19^

The PI3K/AKT pathway plays a key role in tumorigenesis and has been shown to be involved in the regulation of cell growth, differentiation, proliferation, survival and apoptosis. AKT signalling promotes cell proliferation and inhibits apoptotic signalling through a variety of mechanisms.^20^ The activation of AKT signalling pathway regulates cell cycle progression and apoptosis by a variety of mechanisms. First, many intermediate targets, for example GSK3β, FOXO transcription factors and BAD, were phosphorylated by AKT. Second, activation of AKT signalling impeded the inhibition of NFκB, which regulates the transcription of apoptotic and survival genes. Third, active AKT signalling promotes the activation of the downstream effector mTOR by phosphorylation, and mTOR, a serine/threonine kinase, is involved in cell growth, cell cycle progression and survival.^21^

In the latest study, we found that VLX1570 inhibited proliferation, induced apoptosis and ER stress, and blocked the Akt pathway in lung cancer cell lines. Interfering with ER stress by exposure to 4‐phenylbutyric acid (4‐PBA) or tauroursodeoxycholic acid sodium (TUDCA) can fortify the cytotoxicity of VLX1570 in human lung cancer cells. In addition, VLX1570 significantly inhibited the activity of the Akt pathway. Therefore, our data suggest that VLX1570 affected proliferation and apoptosis through ER stress and Akt signalling in human lung cancer cells.

## 2 MATERIALS AND METHODS

### 2.1 Cell culture

A549, H1299 and H460 human carcinoma epithelial‐like cell lines were obtained from the American Type Culture Collection (ATCC), authenticated by STR profiling and tested for mycoplasma contamination by GENEWIZ. All three types of cells were cultured in RPMI 1640 medium (Kai Ji) with 10% foetal bovine serum (FBS) (Bioind) and incubated in a humidified atmosphere with 5% CO_2_, at 37°C.

### 2.2 Cell viability testing

Cell survival was evaluated using a Cell Counting Kit‐8 (CCK‐8) assay (Dojindo) at different drug concentrations. Cells (1 × 10^3^ cells/well) were seeded in 96‐well plates for 24 h and then treated with different doses of VLX1570 (1.25, 2.5, 5, 10, 25, 50, 100, 200 and 400 nM) for 72 h. Afterwards, the medium was removed, and 10 μl of CCK‐8 working solution was added to each well. Cells were then covered with RPMI 1640 and incubated at 37°C for 1 h. The optical density (OD) values of each well were measured at 450 nm using a SpectraMax spectrophotometer (Molecular Devices). The formula:

$$\%\;\text{viability}=\left(\text{ODtest}‐\text{ODblank}/\text{ODcontrol}‐\text{ODblank}\right)\times 100\%$$

was used to calculate half maximal inhibitory concentrations (IC_50_).

### 2.3 Cell proliferation

CCK‐8 assays were used to measure the cell proliferation of A549, H460 and H1299 cells. First, cells (1 × 10^3^ cells/well) were seeded in 96‐well plates for 24 h and then treated with dimethyl sulfoxide (DMSO) or different drug doses of VLX1570 (50, 100, 200 or 400 nM) for 48 h. Next, the medium was removed from each well, and 10 μl of CCK‐8 was added. Cells were maintained at 37°C for 1 h. Finally, the OD values were determined at 450 nm using a SpectraMax spectrophotometer (Molecular Devices). Cell proliferation of combined treatment with VLX1570 and drugs targeting lung cancer was monitored using the Real‐Time xCELLigence Analysis (RTCA, Roche Applied Science and ACEA Biosciences) system.

### 2.4 Western blotting

Protein was extracted from human carcinoma epithelial‐like cell lines using radioimmunoprecipitation assay (RIPA) lysis buffer (Thermo Fisher Scientific). The extracted protein samples were loaded onto 10%–20% sodium dodecyl sulfate polyacrylamide gels and transferred onto polyvinylidene difluoride membranes (Merck Millipore). These membranes were blocked with 5% bovine serum albumin for 1 h and then probed with specific anti‐CyclinB1 (cat. no. ab181593; 1:2000; Abcam, Inc.), anti‐CDK1 (cat. no. ab18; 1:2000; Abcam, Inc.), anti‐CyclinD1 (cat. no. ab134175; 1:30000; Abcam, Inc.), anti‐CyclinE1 (cat. no. ab33911; 1:1000; Abcam, Inc.), anti‐CyclinA2 (cat. no. ab181591; 1:2000; Abcam, Inc.), anti‐Cleaved‐Caspase3 (cat. no. 9661; 1:1000; Cells Signaling Technology, Inc.), anti‐Caspase3 (cat. no. 9662; 1:1000; Cells Signaling Technology, Inc.), anti‐PARP (cat. no. 9532; 1:1000; Cells Signaling Technology, Inc.), anti‐GRP78 (cat. no. 3183; 1:1000; Cells Signaling Technology, Inc.), anti‐IRE1 (cat. no. 3294; 1:1000; Cells Signaling Technology, Inc.), anti‐p‐PERK (cat. no. 3179; 1:1000; Cells Signaling Technology, Inc.), anti‐PERK (cat. no. 5683; 1:1000; Cells Signaling Technology, Inc.), anti‐ATF6 (cat. no. 65880; 1:1000; Cells Signaling Technology, Inc.), anti‐ATF4 (cat. no. 11815; 1:1000; Cells Signaling Technology, Inc.), anti‐p‐eIF2 (cat. no. 5324; 1:1000; Cells Signaling Technology, Inc.), anti‐p‐ERK (cat. no. 4370; 1:1000; Cells Signaling Technology, Inc.), anti‐ERK (cat. no. 4695; 1:1000; Cells Signaling Technology, Inc.), anti‐p‐P38 (cat. no. 4511; 1:1000; Cells Signaling Technology, Inc.), anti‐P38 (cat. no. 8690; 1:1000; Cells Signaling Technology, Inc.), anti‐p‐AKT (cat. no. 4060; 1:1000; Cells Signaling Technology, Inc.), anti‐AKT (cat. no. 4691; 1:1000; Cells Signaling Technology, Inc.), anti‐p‐GSK3 (cat. no. 9323; 1:1000; Cells Signaling Technology, Inc.), anti‐GSK3 (cat. no. 12456; 1:1000; Cells Signaling Technology, Inc.), anti‐p‐mTOR (cat. no. 5536; 1:1000; Cells Signaling Technology, Inc.), anti‐mTOR (cat. no. 2983; 1:1000; Cells Signaling Technology, Inc.), anti‐p‐S6K (cat. no. 9209; 1:1000; Cells Signaling Technology, Inc.), anti‐S6K (cat. no. 2708; 1:1000; Cells Signaling Technology, Inc.), anti‐p‐4EBP1 (cat. no. 2855; 1:1000; Cells Signaling Technology, Inc.), anti‐4EBP1 (cat. no. 9644; 1:1000; Cells Signaling Technology, Inc.) or anti‐GAPDH (cat. no. 5174; 1:1000; Cells Signaling Technology, Inc.) antibodies.

### 2.5 Flow cytometry‐based apoptosis detection

A549, H1299 and H460 cells were seeded in 6‐well plates at a concentration of 2 × 10^5^ cells/well and treated with different doses of VLX1570 (50, 100, 200 and 400 nM) for 24 h. Then, each well was washed with PBS twice, and 500 μl of binding buffer with 5 μl of Annexin V‐FITC propidium iodide (KeyGEN BioTECH) was added, and the plates were kept in the dark. Propidium iodide (PI) was added after 10 min. Cell numbers were determined with a flow cytometer (BD Biosciences) over 1 h and analysed with Modfit software.

### 2.6 Flow cytometry‐based cell cycle analysis

A549, H1299 and H460 cells were seeded in 6‐well plates at a concentration of 2 × 10^5^ cells/well and treated with different doses of VLX1570 (50, 100, 200 and 400 nM) for 24 h. The cells were then washed twice and collected using Trypsin‐EDTA (0.25%) (GIBCO). Afterwards, cells were resuspended in 500 μl of 70% absolute ethyl alcohol at 4°C for at least 12 h. Ultimately, 5 μl of PI solution in the presence of RNase was added to stain cells after permeabilization. A flow cytometer was used to measure the percentage of cells in each part of the cell cycle. Data were analysed using Flowjo software.

### 2.7 Colony formation assays

Cells were collected and seeded at a density of 1000 cells per well in 6‐well plates. After 24 h of culture, A549, H460 and H1299 cells were washed with PBS three times and then fixed with 4% paraformaldehyde phosphate buffer. Finally, 5% crystal violet was used to stain and visualize cell colonies. The number of colonies was recorded and counted to evaluate cell proliferation abilities.

### 2.8 Quantitative real‐time PCR (RT‐qPCR)

The whole RNA from cells was extracted using Takara MiniBEST Universal RNA Extraction Kit (Takara Biotechnology CO., Ltd). Reverse transcriptions were performed by PrimerScript RT Master mix (Takara Biotechnology CO., Ltd) after the concentration and quality of total RNA were determined by 260/280 nm absorbance. ABI 7300 PCR system (Applied Biosystems) was applied to carry out the quantitative PCR reaction by using a SYBR Green Master Mix (Thermo Fisher Science). Gene‐specific primers and GAPDH were designed and synthesized by Sangon Biotechnology Co. (Sanghai, China) according to the gene sequences in Genbank. The primer sequences were as follows: CDK1 (forward primer, 5′‐CACAAAACTACAGGTCAAGTGG‐3′ and reverse primer, 5′‐TCAGATCCATGGAAAGAAACTCA‐3′), Cyclin B1 (forward primer, 5′‐GACTTTGCTTTTGTGACTGACA‐3′ and reverse primer, 5′‐CCCAGACCAAAGTTTAAAGCTC‐3′), GAPDH (forward primer, 5′‐GGATTTGGTCGTATTGGGCG‐3′ and reverse primer, 5′‐ATCGCCCCACTTGATTTTGG‐3′). All experiments were accomplished three times, and relative expression was normalized to GAPDH as an internal control and calculated using 2^−△△^CT.

### 2.9 Statistical analysis

All independent experiments were performed in triplicate. Data are presented as means ± standard deviation (SD). Comparisons between two groups were analysed with Student's *t* test, while comparisons among multiple groups were conducted using multiple *t* tests. Statistical analysis was performed with GraphPad Prism 8.0. The criterion for statistical significance was *p* < 0.05.

## 3 RESULTS

### 3.1 VLX1570 is a specific inhibitor targeting lung cancer cells

**FIGURE 1:**
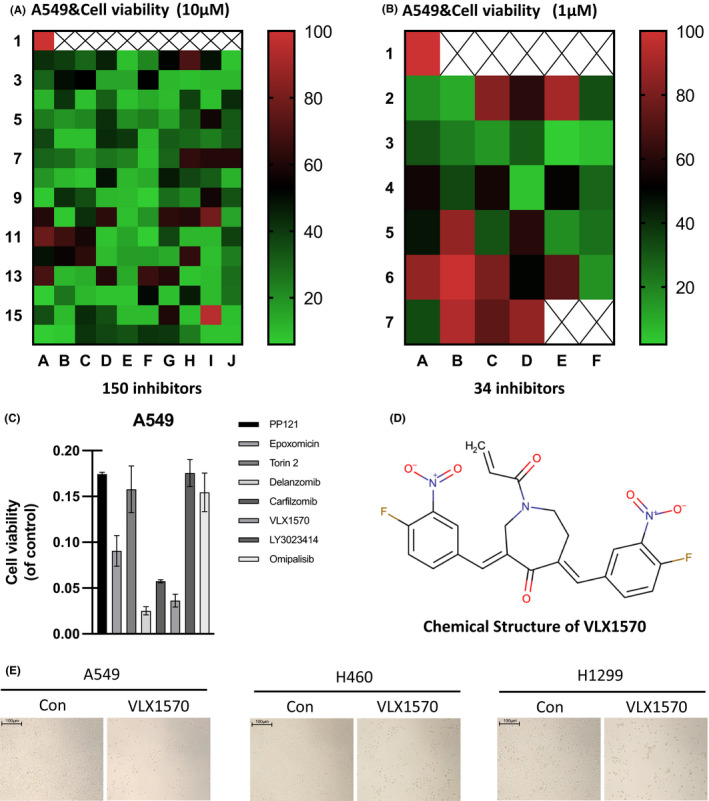
VLX1570 is a specific inhibitor targeting lung cancer cells. (A) The heat map illustrates the results of the initial screening of 150 inhibitors in 10 μM. (B) The heat map illustrates the results of the re‐screening of 34 inhibitors in 1 μM. (C) The survival rate of the eight most effective inhibitors. (D) The chemical structure of VLX1570. (E), Bright‐field image of lung cancer cells treated with VLX1570 (1 μM) for 96 h (scale bar, 100 μM)

Initially, we completed a large‐scale screening of 150 small molecules (at a concentration of 10 μM) using a Ubiquitination Compound Library purchased from Selleck, with the goal of identifying novel compounds that were selectively toxic to lung cancer cells (Figure 1A). The survival rates of lung cancer cells treated with each drug molecule are plotted in a heat map shown in Figure 1A. We then selected 34 molecules with survival rates less than 20% from the preliminary screening results for re‐screening at 1 μM (Figure 1B). From the results of this re‐screening, we identified eight of the most effective molecules (Figure 1C). Our results showed that Delanzomib and VLX1570 had the strongest inhibition effects (Figure 1C). Delanzomib has been reported to inhibit the proliferation of lung cancer cells.^22^ Thus, we chose VLX1570 for further study in lung cancer cells. Figure 1D shows the chemical structure of VLX1570.

VLX1570 can dramatically kill lung cancer cells in a low drug concentration (Figure 1E).

### 3.2 VLX1570 suppresses the growth and proliferation of human lung cancer cells

**FIGURE 2:**
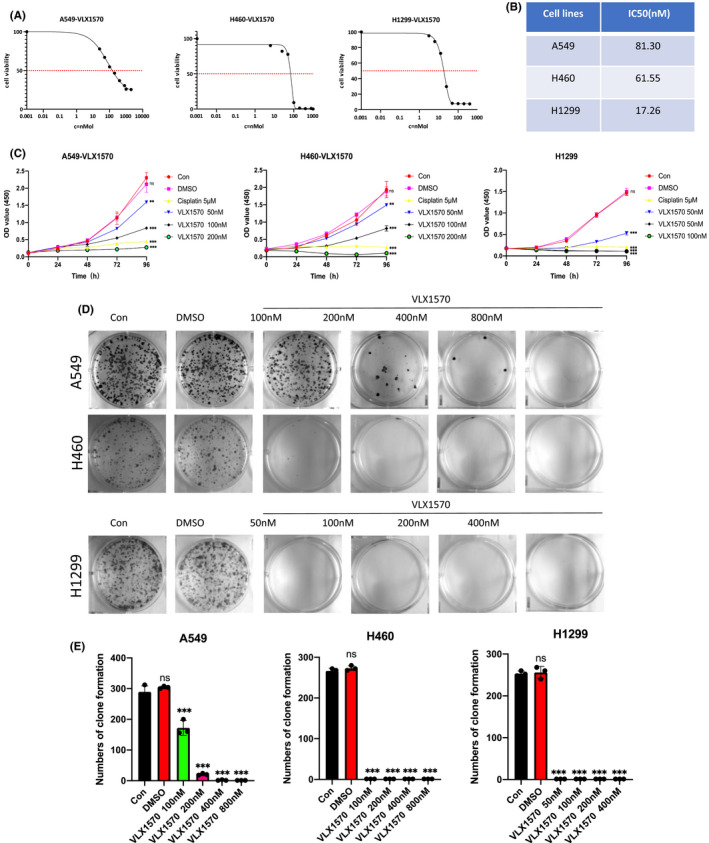
VLX1570 suppresses the proliferation of human lung cancer cells. (A,B) The IC50 of VLX1570 in A549, H460 and H1299 cells. (C), Dose‐viability curves of A549, H460 and H1299 cells treated with different concentration of VLX1570 for 0, 24, 48, 72 and 96 h. (D), A549, H460 and H1299 cells were cultured in 6‐well plates. VLX1570 (50, 100, 200, 400 and 800 nM) or vehicle (DMSO) were added to culture media, and colony forming efficiency was determined 10–14 days later. (E), The data represent the mean ± SD from three independent experiments, **p < 0.01, ***p < 0.001

Next, A549, H460 and H1299 lung cancer cells were treated with varying doses of VLX1570 for 72 h, and CCK‐8 was used to determine the cell viability rates (Figure 2A). The IC_50_ of A549, H460 and H1299 cells were shown after treatment with VLX1570 (Figure 2B). Then, the three kinds of cells were treated with different doses of VLX1570 for 0, 24, 48, 72 and 96 h (Figure 2C). The outcome demonstrated that VLX1570 inhibited the cell proliferation of human lung cancer cells in a concentration‐ and time‐dependent manner (Figure 2C). In addition, the numbers of colonies formation, among each dosage group, were significantly decreased compared with the control group (Figure 2D,E). In total, our results indicated that VLX570 suppressed the growth of lung cancer cells in a concentration‐ and time‐dependent manner.

### 3.3 VLX1570 arrested cells in G2/M phase by decreasing CyclinB1 and CDK1 levels

**FIGURE 3:**
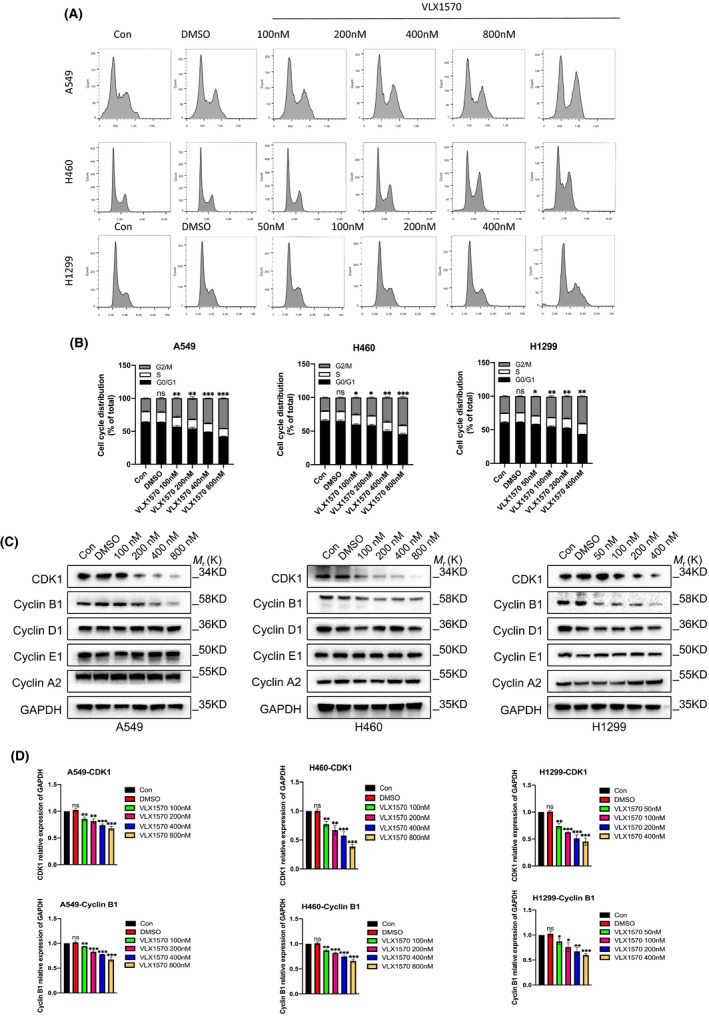
VLX1570 arrests cell cycle at G2/M phase in lung cancer cells. (A) The distribution of cell cycle after VLX1570 treatment and quantitative analyses were examined by flow cytometry in A549, H460 and H1299. (B) The data represent the mean ± SD from three independent experiments, *p < 0.05, **p < 0.01. (c) The expression levels of multiple cell‐cycle‐associated proteins were assessed by Western blot assay in does‐manner. (d) The transcriptional level of CDK1 and CyclinB1 was assessed by QPCR assay in dose‐dependent manner. The bars represent the mean ± SD from three independent experiments. *p < 0.05, **p < 0.01

We then tested the effect of VLX1570 on the cell cycle. Human lung cancer H460 and H1299 cells were treated with different doses of VLX1570 for 24 h. Then, the cell cycle profiles of VLX1570‐treated H460 and H1299 cells were determined by flow cytometry (Figure 3A). Both cell types remained largely in G2/M phase when treated with VLX1570 (Figure 3B). The results showed that VLX1570 arrested cells in G2/M phase. To investigate how VLX1570 regulates the cell cycle, we measured cell cycle‐related proteins by Western blot. Indeed, among the several cell cycle‐related proteins investigated, CyclinB1 and CDK1 were noticeably decreased upon VLX1570 treatment in a dose‐dependent manner in lung cancer cells (Figure 3C). This result revealed that VLX1570 significantly inhibited the expression of Cyclin B1 and CDK1. At the same time, VLX1570 did not change the levels of Cyclin D1, Cyclin A2 or Cyclin E1, which validated the notion that VLX1570 could cause a G2/M phase block *in vitro* (Figure 3C). Then, we detected the transcriptional level of CDK1 and CyclinB1. This result revealed that VLX1570 significantly downregulated the transcriptional level of CDK1 and CyclinB1 (Figure 3D). Our results indicated that VLX1570 arrested the cell cycle at G2/M phase through promoting the degradation of CyclinB1 and CDK1 in lung cancer cells.

### 3.4 VLX1570 induces cell apoptosis via the mitochondrial‐associated pathway in human lung cancer cells

**FIGURE 4:**
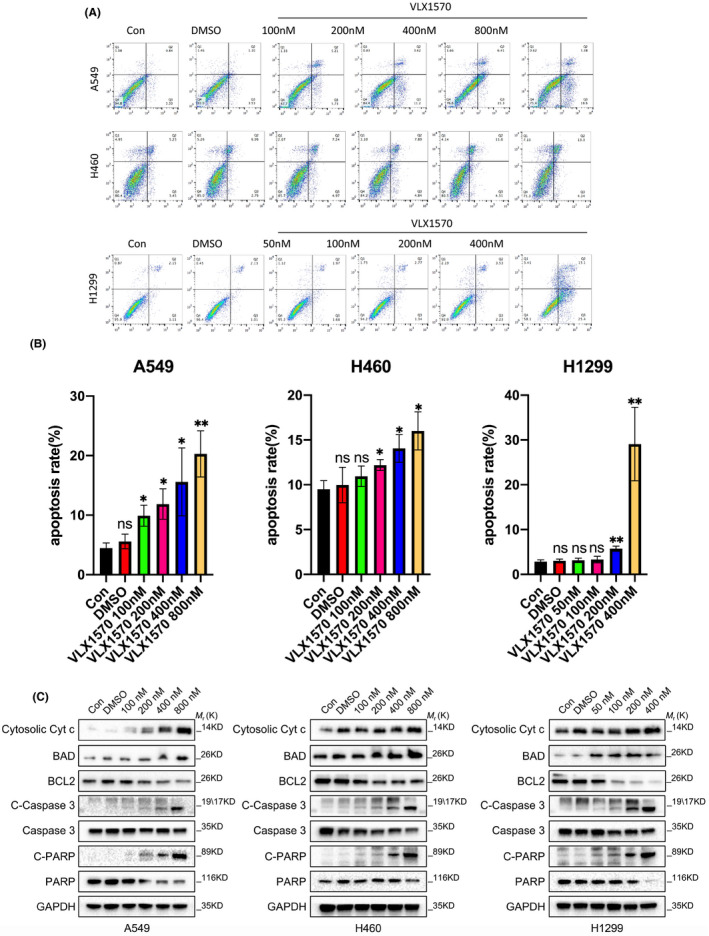
VLX1570 induces mitochondrial‐associated cell apoptosis in human lung cancer cells. (A) A549, H460 and H1299 cells were treated with different dose of VLX1570 for 24 h, respectively. The apoptotic cell population including early and late apoptosis cells was quantified using an apoptotic Kit and analysed by flow cytometry. (B) Bars indicate the frequency of apoptotic cell population including early and late apoptosis cells. (C) A549, H460 and H1299 cells were treated with different dose of VLX1570 for 24 h, respectively. Lysates were blotted with the indicated antibodies. The bars represent the mean ± SD from three independent experiments. *p < 0.05, **p < 0.01

Next, we aimed to ascertain the influence on VLX1570‐induced apoptosis in human lung cancer cells after treatment with VLX1570. Different doses of VLX1570 were used to treat A549, H460 and H1299 cells for 24 h. Compared with controls, the apoptotic rate was obviously increased in A549, H460 and H1299 cells following treatment with different concentrations of VLX1570, as detected by flow cytometric analysis (Figure 4A,B). We then explored the expression of apoptosis‐associated proteins. Our results demonstrated that VLX1570 remarkably promoted the levels of cleaved PARP and cleaved caspase3 in all three cell lines in a concentration‐dependent manner (Figure 4C). In addition, VLX1570 upregulated the level of cytosolic cytochrome c and BAD while downregulating BCL2 in A549, H460 and H1299 cells (Figure 4C). These results proved that the apoptosis of A549, H460 and H1299 cells were significantly induced by VLX1570 through the mitochondrial apoptotic pathway.

### 3.5 ER stress is involved in VLX1570‐induced apoptosis in human lung cancer cells

**FIGURE 5:**
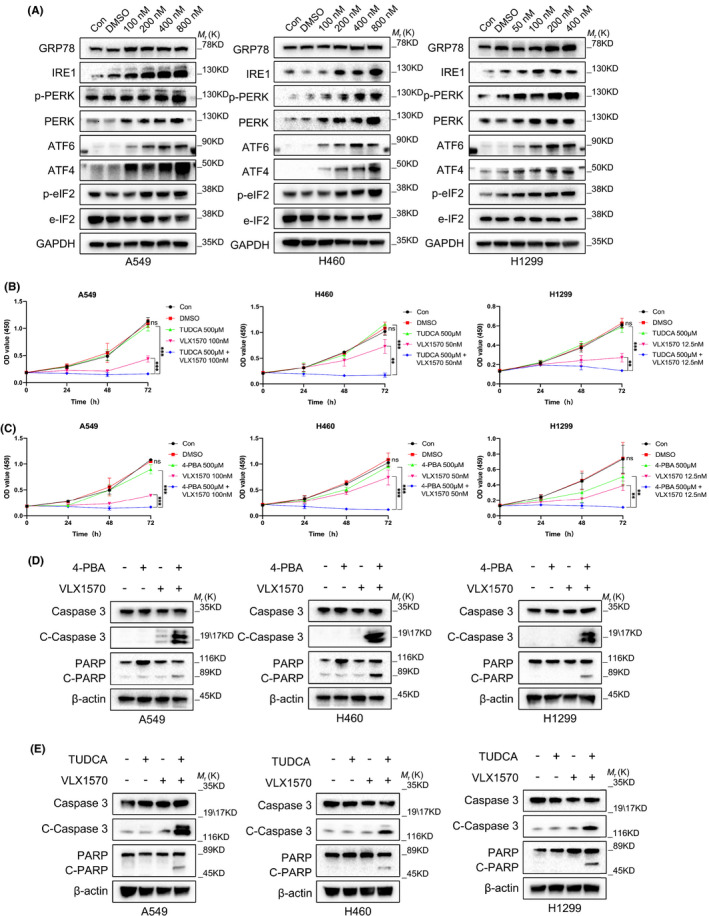
Apoptosis caused by VLX1570 is related to ER stress‐associated pathway. (A) A549, H460 and H1299 cells were treated with 50, 100, 200, 400 and 800 nM VLX1570 for 24 h. Western blotting analysis of the expression of GRP78, ER stress‐associated apoptosis proteins ATF6, PERK and IRE1. (B, C) Inhibition of ER stress fortified the cytotoxicity of VLX1570 in A549, H460 and H1299 cells. (D, E) The level of cleaved PARP and cleaved caspase3 proteins in A549, H460 and H1299 cells treated with VLX1570 and/or 4‐PBA for 24 h were determined by Western blotting

Previous studies have demonstrated that VLX1570 induces cell death by triggering ER stress.^19^ Therefore, we further explored whether VLX1570 induced ER stress in human lung cancer cells. In order to affirm this, we first determined the expression levels of GRP78 and ER stress‐associated proteins in A549, H460 and H1299 cells (Figure 5A). ER stress could trigger the UPR to respond to environmental factors when it was induced. The UPR regulates the expression of the ER stress sensor proteins PERK, IRE1 and ATF6. As shown in Figure 5A, the expression levels of GRP78, p‐PERK, IRE1, ATF6, ATF4 and p‐eIF2α were increased in all three human lung cancer cells in a dose‐dependent manner (Figure 5A). All these consequences indicated that ER stress was induced in A549, H460 and H1299 cells treated with VLX1570, which was controlled by the PERK, IRE1 and ATF6 pathways.

Next, we examined the correlation of ER stress in VLX1570‐induced apoptosis in human lung cancer cells. ER stress inhibitors, 4‐PBA and TUDCA, were used to alleviate ER stress in the A549, H460 and H1299 cells treated with VLX1570. We noticed that the cell viability of the VLX1570‐treated A549, H460 and H1299 cells was effectively reduced after the exposure to inhibition of ER stress at 24, 48 and 72 h through CCK‐8 assay (Figure 5B,C). Then, we detected the level of apoptosis‐associated proteins, cleaved PARP and cleaved caspase3. Our results manifested that activation of PARP and caspase3 was significantly upregulated after being treated with VLX1570 and ER stress inhibitors simultaneously (Figure 5D,E). Inhibition of ER stress remarkably facilitate the cell apoptosis of VLX1570‐treated A549, H460 and H1299 cells.

### 3.6 VLX1570 blocks the AKT pathway

**FIGURE 6:**
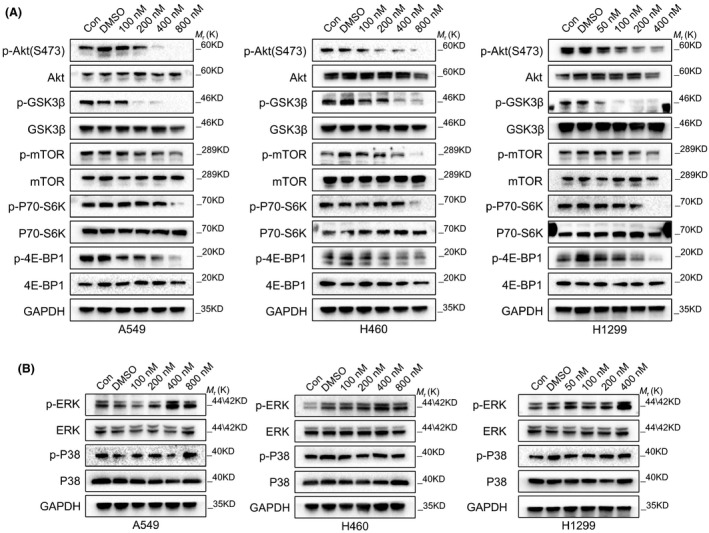
VLX1570 inhibit the AKT pathway. (A) A549, H460 and H1299 cells were treated with different dose of VLX1570 for 24 h, respectively. The cell lysates were subjected to analysis of the phosphorylation levels of Akt and its downstream targets. (B) The indicated cell lysates were subjected to analysis of the phosphorylation levels of ERK and p38

The AKT pathway is associated with multiple functions, such as cell apoptosis, proliferation, survival, metastases invasion and tumorigenesis, in lung cancer.^23^ Inhibition of the AKT pathway induces cell cycle arrest and suppresses cell growth.^24^ However, it is unclear whether VLX1570 had any effect on the Akt pathway. We treated lung cancer cells with different concentrations of VLX1570 and detected molecular changes in the Akt pathway. VLX1570 decreased the phosphorylation levels of Akt and Akt downstream targets such as GSK3β, mTOR, p70‐S6K and 4E‐BP1, without affecting their total protein levels (Figure 6A). In addition, VLX1570 had no effect on the ERK pathway (Figure 6B). These results suggested that VLX1570 may regulate the proliferation, cell cycle, and apoptosis of lung cancer by specifically inhibiting the Akt pathway.

### 3.7 Combined treatment with VLX1570 and drugs targeting lung cancer induces synergistic anti‐lung cancer activity

Afatinib is one of a new generation of oral small molecule tyrosine kinase inhibitors. It is the first irreversible ErbB family blocker and can act on the entire ErbB family including EGFR. Gefitinib, as a small molecule compound, is an oral epidermal growth factor receptor tyrosine kinase (EGFR‐TK) inhibitor. EGFR is one of the most common mutated genes in NSCLC, and in Asians, it accounts for 59.7% of cases.^25^ Although the response rate of EGFR‐mutated NSCLC patients is around 75% after treatment with Afatinib and Gefitinib.^26^, ^27^ Unavoidably, patients still progress as drug resistance mechanisms develop.^28^

**FIGURE 7:**
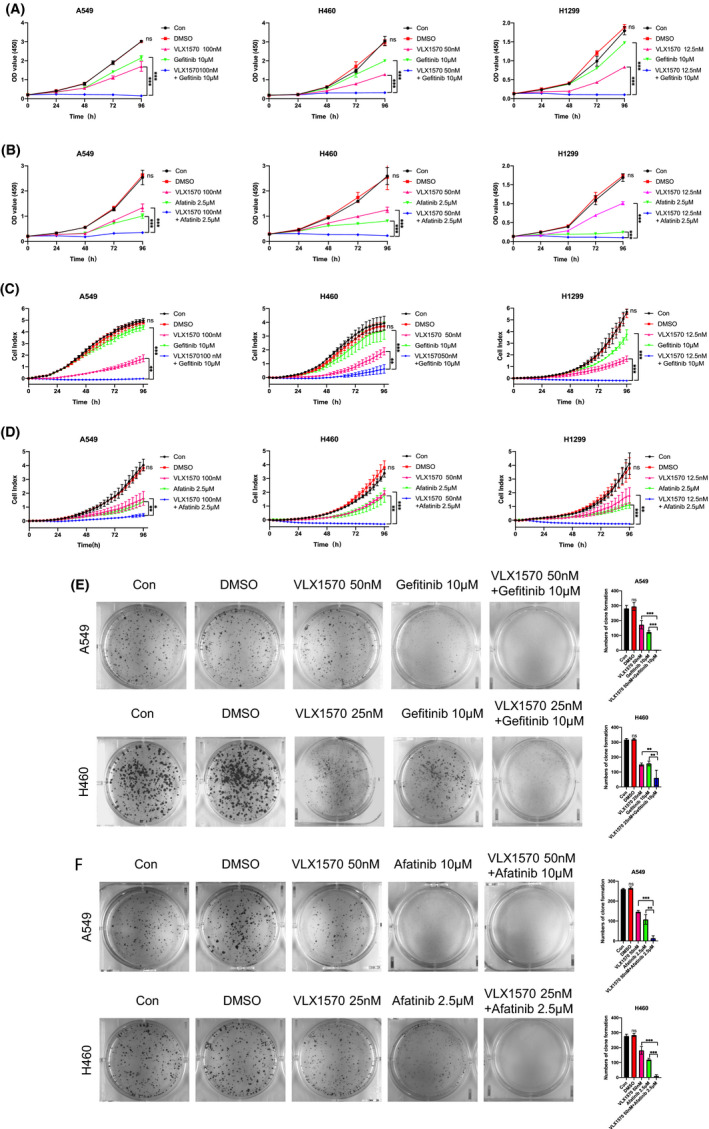
Combined treatment with VLX1570 and Afatinib or Gefitinib induces synergistic anti‐lung cancer activity. (A, C, E) A549, H460 and H1299 cells were treated for different time with VLX1570, Gefitinib or VLX1570 plus Gefitinib and then assessed for viability using CCK‐8 assays, RTCA and colony formation. The synergistic cytotoxic impact of VLX1570 and Gefitinib was showed in isobologram analysis. (B, D, F) A549, H460 or H1299 cells were treated for different time with VLX1570, Afatinib or VLX1570 plus Afatinib and then assessed for viability using CCK‐8 assays, RTCA and colony formation. The synergistic cytotoxic impact of VLX1570 and Gefitinib was showed in isobologram analysis

We tested the effects of combining treatments including Gefitinib and Afatinib at lower 3, with or without VLX1570, on lung cancer cell viability. Overall, at low concentrations of Gefitinib, Afatinib and VLX1570, these combined treatments tended to cause a greater reduction in cell viability as compared to the effect of single agents at corresponding concentrations (Figure 7A,B). Moreover, cell proliferation was measured using the RTCA system and colony formation assays (Figure 7C–F). These experiments showed that the proliferative ability was attenuated when combined treatment with VLX1570. Synergistic anti‐lung cancer activity demonstrated that the combination of low concentrations of VLX1570 and targeted drugs triggered synergistic anti‐lung cancer activity. In summary, these results indicated that combined treatments tended to cause a greater reduction in cell viability as compared to the effect of single agents at corresponding concentrations.

## 4 DISCUSSION

**FIGURE 8:**
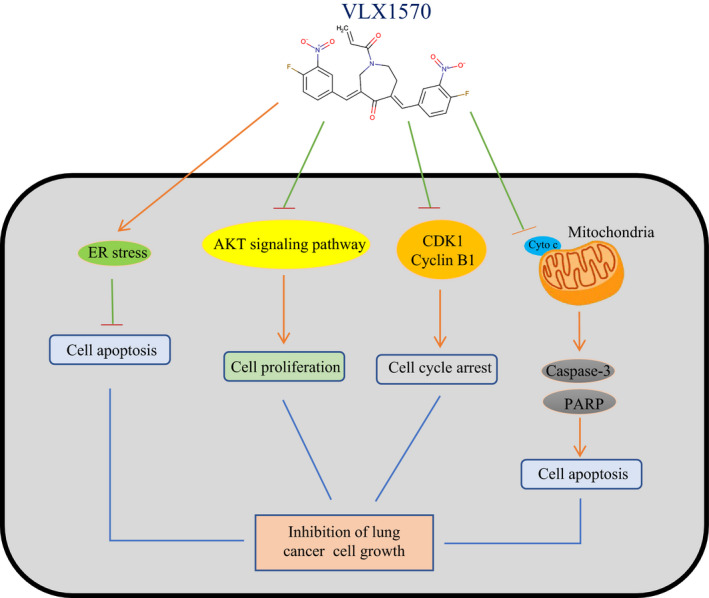
Schematic diagram of the molecular mechanisms by VLX1570 to regulate the growth of lung cancer cells

Lung cancer is still one of the most common malignancies and often becomes resistant to many chemotherapeutic regimens, which represents a significant obstacle to clinical treatment. The UPS is an enzyme network, which has been studied for nearly 40 years since it was firstly discovered in 1975. Ubiquitination, as an important post‐translational modification (PTM), can regulate a large amount of signalling pathways and participate in many biological processes.^5^ Due to the importance of the UPS in normal biological processes, its changes often lead to the aetiology of many diseases, especially cancer.^29^ Based on the phenomenon that abnormal UPS activity often happened in human cancers, potential therapeutic targets have been identified, and corresponding inhibitors have been developed.^30^ At present, the proteasome is a successful clinical target, and some FDA‐approved drugs have also achieved good therapeutic effects, such as bortezomib, carfilzomib, oprozomib and ixazomib.^31^ However, as the last step in the ubiquitination process, these drugs can cause some side effects due to the accumulation of upstream ubiquitinated proteins, which limits their widespread application.^31^ Therefore, researchers endeavour to develop targeted inhibitors of DUBs. DUB inhibitors are expected to overcome the obstacles in the clinical treatment of lung cancer (Figure 8).

Several effective inhibitors against USP7 have been developed, most of which show high specificity for USP7. P5091, a dose‐dependent ubiquitin‐competitive USP7 inhibitor, can specifically block the binding between ubiquitin and the USP7 catalytic domain.^32^ All USP14 inhibitors are non‐selective inhibitors because other 19S proteasome‐associated DUBs such as UCHL5 have shared structural similarity with USP14.^33^ b‐AP15 is typical of the USP14 inhibitors. VLX1570 is an analog of b‐AP15, which has higher potency and solubility than b‐AP15. It is also a reversible non‐selective competitive inhibitor of USP14, targeting the formation of Ub‐USP14 or Ub‐UCHL5 conjugates.^32^ This is achieved by inhibiting the enzymatic activity of USP14 and UCHL5, and VLX1570 shows significant anti‐cancer impact in multiple myeloma and Waldenstrom's macroglobulinemia.^32^ Anchored in these findings, a phase 1/2 trial evaluating the efficacy and tolerability of VLX1570 in patients with relapsed or refractory multiple myeloma is currently under way (NCT02372240).^31^

ER stress can trigger the UPR, which regulates and controls the expression of the ER molecular chaperone GRP78/Bip, the ER stress sensor protein PERK and inositol‐requiring enzyme 1 (IRE‐1). Mild–to‐moderate ER stress accelerates cell proliferation through the UPR to alleviate ER stress.^34^ However, persistent and severe ER stress or inhibition of ER stress can result in cell death in some cancers including lung cancer. The specific function of VLX1570‐induced ER stress is still unclear. Previous studies have demonstrated that ER stress accelerates tumour angiogenesis and vascular regeneration.^35^ Prolonged ER stress activation induces apoptosis,^36^ and apoptosis caused by ER stress is transduced by the expression of pro‐apoptotic proteins.^37^ In the previous study, researchers demonstrated that VLX1570 can result in ER stress through the expression of GRP78, IRE1 and PERK.^19^ Cytotoxicity and apoptosis were remarkably fortified in human lung cancer A549 and H460 cells treated with VLX1570 when we blocked ER stress using the ER stress inhibitors 4‐PBA or TUDCA.

Activating the AKT pathway triggers the phosphorylation of GSK3β, which regulates glycogen synthase; then, through a series of reactions this pathway inhibits the phosphorylation of the downstream effector mTOR, a serine/threonine kinase, that is associated with cell proliferation, cell cycle progression and angiogenesis.^38^ The proteins RPS6KB1 (70 kDa, polypeptide 1, also called S6K) and EIF4E‐BP1 (also called 4E‐BP1), which take part in cell proliferation and may be useful as activity pathway markers in human tumours, are the two most relevant downstream effectors of Mtor.^39^ Taking together, finding targeted therapies for AKT/mTOR downstream effectors is essential for the treatment of lung cancer. In our study, VLX1570 was shown to inhibit the activity of the Akt pathway. VLX1570 inhibits the proliferation of lung cancer cells, causes cell cycle arrest and promotes cell apoptosis by inhibiting the Akt pathway. The mechanism of VLX1570 regulation of Akt, however, needs to be further studied. In addition, combined treatment with VLX1570 and Gefitinib or Afatinib induces synergistic anti‐lung cancer activity. In conclusion, VLX1570 may provide a novel method for the treatment of lung cancer and has great clinical application value.

## 5 CONCLUSION

Taken together, the results of this study showed that VLX1570 significantly inhibited tumour cell colony formation, induced cell cycle arrest and inhibited lung cancer cell proliferation *in vitro*. Moreover, we found that VLX1570 downregulated CyclinB1 and CDK1 to induce cell cycle arrest. Moreover, VLX1570 upregulated cleaved PARP, cleaved caspase3 and cytosolic cytochrome c to induce apoptotic cell death in human lung cancer cells, indicating that VLX1570 can serve as an anti‐cancer drug for lung cancer. Aside from this, we found that VLX1570 regulated ER stress‐induced apoptosis and led to the inactivation of the Akt pathway in human lung cancer cells. This study uncovered the potential molecular mechanisms driving VLX1570‐induced lung cancer cell death and provides a theoretical basis for the application of VLX1570 for lung cancer treatment in a clinical setting.

## CONFLICT OF INTEREST

The authors confirm that there are no conflicts of interest.

## AUTHOR CONTRIBUTIONS

Jifeng Feng: Conceptualization (equal); Funding acquisition (equal). Juan Wang: Data curation (lead). Tongde Du: Data curation (equal); Funding acquisition (equal). Ya Lu: Data curation (supporting). Yan Lv: Data curation (supporting). Yuxin Du: Data curation (supporting). Jianzhong Wu: Formal analysis (supporting); Funding acquisition (equal). Rong Ma: Methodology (supporting). Chenxin Xu: Formal analysis (supporting).

## Data Availability

The data used to support the findings of this study are available from the corresponding author upon request.
